# Supplementary material for: Adding Value in Production of Multifunctional Polylactide (PLA)–ZnO Nanocomposite Films through Alternative Manufacturing Methods
Source: Molecules. 2021 Apr 2;26(7):2043. doi: 10.3390/molecules26072043 (PMC8038199; doi:10.3390/molecules26072043)
Supplement: Supplementary file 1 [file molecules-26-02043-s001.pdf]

Supplementary Material S1 (online publication)

Table S1. Comparative molecular parameters determined by SEC* of PLA - ZnO nanocomposites extruded as films using different methods.

Sample	Method	$M_{n(PS)}$	$M_{w(PS)}$	PI
PLA (reference)	-	113 600	205 800	1.81
PLA- 1% $ZnO_{(MB)}$	A	92 100	194 000	2.11
PLA- 1% ZnO	B	69 700	149 600	2.15
PLA- 3% $ZnO_{(MB)}$	A	92 600	195 800	2.11
PLA- 3% ZnO	B	54 800	117 500	2.14

*Size exclusion chromatography (SEC)

Molecular weight determination of PLA was carried out on samples dissolved in chloroform (10 mg polymer/5 ml solvent). Size exclusion chromatography (SEC) measurements on previous centrifugated and filtered samples were performed at 30°C using an Agilent liquid chromatograph equipped with an Agilent degasser, an isocratic HPLC pump (flow rate = 1 mL/min), an Agilent autosampler (loop volume = 100 µL), an Agilent-DRI refractive index detector and three columns: a PL gel 5 µm guard column and two PL gel Mixed-B 5 µm columns (linear columns for separation of molecular weights (PS standards) ranging from 200 to 4.10^5 g/mol). The experimental errors in the case of SEC measurements are estimated to about 10%. Molecular weights and molecular distributions were calculated by reference to a universal calibration curve relative to polystyrene standards.

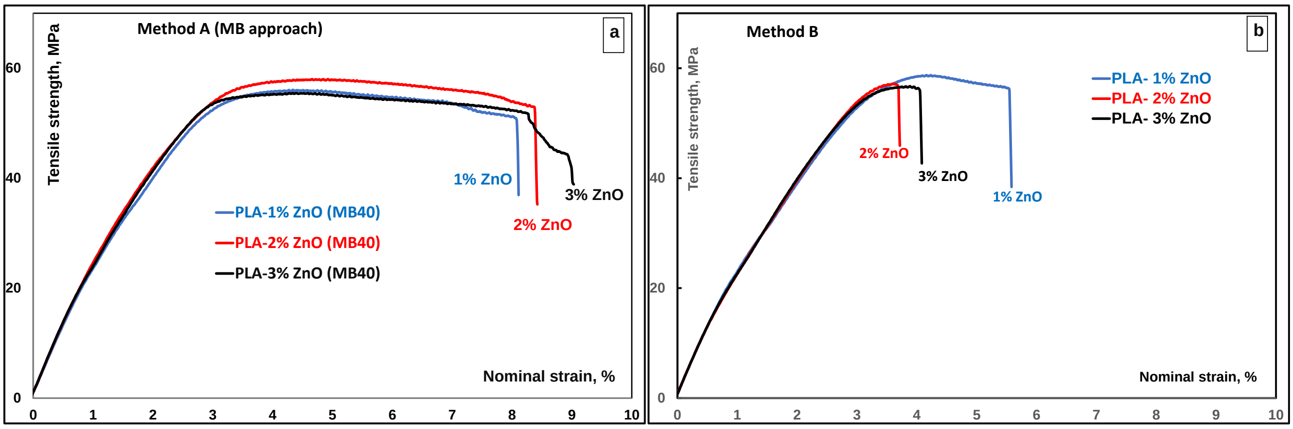

Figure S1 (a, b). Typical tensile stress – strain curves of PLA - ZnO nanocomposite films (thickness ~400 μm) produced using **a)** the MB approach (method A) and **b)** PLA – ZnO nanocomposites as granules (method B, current technique.
